# Calcium Signalling in Medulloblastoma: An In Silico Analysis of the Expression of Calcium Regulating Genes in Patient Samples

**DOI:** 10.3390/genes12091329

**Published:** 2021-08-27

**Authors:** Ahmed Maklad, Mohammed Sedeeq, Michael J. G. Milevskiy, Iman Azimi

**Affiliations:** 1 School of Pharmacy and Pharmacology, College of Health and Medicine, University of Tasmania, Hobart, TAS 7005, Australia; ahmed.maklad@utas.edu.au (A.M.); mohammed.sedeeq@utas.edu.au (M.S.); 2 ACRF Cancer Biology and Stem Cells Division, The Walter and Eliza Hall Institute of Medical Research, Parkville, VIC 3052, Australia; milevskiy.m@wehi.edu.au; 3 Department of Medical Biology, The University of Melbourne, Parkville, VIC 3010, Australia

**Keywords:** calcium signalling, medulloblastoma, gene expression, in silico analysis

## Abstract

Dysregulation in calcium signalling is implicated in several cancer-associated processes, including cell proliferation, migration, invasion and therapy resistance. Modulators of specific calcium-regulating proteins have been proposed as promising future therapeutic agents for some cancers. Alterations in calcium signalling have been extensively studied in some cancers; however, this area of research is highly underexplored in medulloblastoma (MB), the most common paediatric malignant brain tumour. Current MB treatment modalities are not completely effective and can result in several long-lasting mental complications. Hence, new treatment strategies are needed. In this study, we sought to probe the landscape of calcium signalling regulators to uncover those most likely to be involved in MB tumours. We investigated the expression of calcium signalling regulator genes in MB patients using publicly available datasets. We stratified the expression level of these genes with MB molecular subgroups, tumour metastasis and patient survival to uncover correlations with clinical features. Of particular interest was CACNA1 genes, in which we were able to show a developmentally-driven change in expression within the cerebellum, MB’s tissue of origin, highlighting a potential influence on tumour incidence. This study lays a platform for future investigations into molecular regulators of calcium signalling in MB formation and progression.

## 1. Introduction

Calcium ions (Ca^2+^) are the most abundant second messengers in the human body, where they play vital roles in various physiological processes. Disruption in calcium signalling can cause defects in normal cell growth and is linked to cancer initiation, proliferation and invasion [1]. Ca^2+^ plays an integral role in cell cycle progression where it controls the induction of immediate response genes such as FOS, JUN and MYC. Ca^2+^ is vital in G1/S and G2/M transitioning, where depletion of extracellular Ca^2+^ store can lead to cell cycle cessation [1]. Dysregulated expression of calcium influencing cell cycle proteins has been implicated in several cancers [2,3,4]. The role of Ca^2+^ in malignant cell migration and metastasis has been extensively elucidated in recent years. In general, the mechanisms by which cancer cells migrate do not appear to be different from those found in normal physiological migration; however, the magnitude of the events is the primary difference [5]. In many instances, migrated cancer cells show aberrant expression of calcium controlling genes leading to a higher activity of key invasion markers such as matrix metalloproteinases, MMP2 and MMP9 [6]. Calcium is a key player in the phosphorylation of contractile proteins required for maintaining the morphological changes for efficient cell migration [7]. Yang et al. reported evidence for the role of STIM1 and ORAI1 in the metastasis of breast cancer. This study showed that the level of STIM1 expression is directly associated with metastasis and reduced survival among breast cancer patients, and blocking store-operated calcium entry reduces the migration of breast cancer cells [8]. Transient receptor potential channel (TRP) is another group of calcium controlling genes that have been heavily investigated in cancer cell migration [5,7,9]. TRPM7, for example, has been shown to regulate cell migration, through myosin II-dependent contractility, in human breast cancer cells [10], and TRPV1 is linked to the modulation of migration and invasion of several cancer types [11].

Medulloblastoma (MB) is the most common paediatric malignant brain tumour accounting for about one-fifth of all childhood brain cancers. MB is classified into four molecular subgroups of Wingless (WNT), Sonic Hedgehog (SHH), Group 3 and Group 4. The molecular subgroups of MB directly influence the prognostic variability between MB patients [12]. While there has been quite an advancement in the treatment of WNT and SHH subgroups with a 5-year overall survival rate of over 90% and around 80% respectively [13], Groups 3 and 4 MBs remain a challenge. Indeed, these subgroups account for >60% of all MBs, are only seen in children and are associated with worse clinical outcomes compared with the other two groups [13].

Current treatments available for MB involve surgical resection to remove the tumour, radiotherapy and chemotherapy. These treatment modalities are not highly effective and can result in many complications as they often target highly proliferative cells that when affected in the developing brain of children have devastating long-lasting effects that include neurological, intellectual and physical disabilities [14]. It is therefore essential to explore new therapeutic options with improved treatment efficacy and specificity for the underlying biology of MB. Despite the increasing evidence for the role of calcium signalling in tumour development and invasion in many types of cancers, few studies have investigated calcium signalling in MB. One such study by Wei et al. demonstrated that activation of the TRPC4 calcium channel resulted in an increase in Ca^2+^ influx and enhancement of MB cell motility, highlighting the importance for more focus on this research [15].

In this study, we investigated the gene expression of a panel of calcium signalling regulators including channels, pumps, receptors and sensors in MB patients using publicly available patient datasets. We explored the correlation in the expression of these genes with MB molecular subgroups, tumour metastasis and patient survival. This study is the first to systematically identify potential calcium regulators in MB that may show involvement in tumour progression and present as potential therapeutic targets.

## 2. Materials and Methods

### 2.1. Analysis Platforms and Patient Datasets

R2 Genomics Analysis and Visualization Platform (http://r2.amc.nl (accessed on July 2020)) and GlioVis data portal for visualisation and analysis of brain tumour expression datasets [16] were used to extract gene expression data. Datasets used in this study include Pomeroy [17], Donson [18], Zhao [19], and Cavalli [20]. Table 1 summarises the patient samples, including tissues from normal brain and MB tumours and the sequencing platform used to generate expression data.

### 2.2. Expression Analysis in MB and Normal Brain Tissues

Pomeroy [17], Donson [18], and Zhao [19] datasets were used to assess gene expression in normal brain and MB tissues with a *p*-value of less than 0.01 set as a cut-off for statistical significance. Data were extracted from the R2 platform and plotted in GraphPad Prism Version 9.1 software for Windows (La Jolla, CA, USA). The Pomeroy dataset was used to assess gene expression in tissues from the normal brain and four MB subgroups. To generate a gene expression heatmap stratified by tumour subtype, values were normalised by mean-centring.

### 2.3. Expression Analysis in GTML Mouse Model

Transcriptomic data of 5 normal cerebellum and 32 MB tumours from the GTML mouse model were sourced from the National Center for Biotechnology Information’s Gene Expression Omnibus (GEO) (accession number GSE36594, [21]). Data were analysed using the GEO2R online tool available at http://www.nci.nlm.nih.gov/geo/geo2r/ (accessed on May 2021). Expression values are presented as log 2 RMA values.

### 2.4. Expression Analysis in Brain Sections

For analysis of different brain sections, microarray data from 6 brain samples (donor IDs: 9861, 10,021, 12,876, 14,380, 15,496, and 15,697) were obtained from the Allen Human Brain Atlas dataset (https://human.brain-map.org/ (accessed on May 2021)). Expression values of CACNA1H (probe name A_23_P26294), CACNA1A (probe name A_24_P130559) and CACNA1D (probe name A_23_P365767) are presented in log2.

Microarray data for analysis of the cerebellum of the developing brain were obtained from BrainSpan Atlas of the Developing Human Brain (www.brainspan.org (accessed on May 2021)). A total of 31 cerebellum samples of individuals from 12 post-conceptual weeks to 40 years were used (donor ID for each sample is stated in the respective figure). Expression values of CACNA1H (Ensembl ID ENSG00000196557), CACNA1A (Ensembl ID ENSG00000141837) and CACNA1D (Ensembl ID ENSG00000157388) are presented in reads per kilobase of transcript (RPKM).

### 2.5. Correlation of Gene Expression Levels with Tumour Metastasis

Gene expression and metastasis stratification was conducted using the Cavalli dataset [20], where the metastatic status of patients at diagnosis is recorded. Expression values were shown as log 2.

### 2.6. Stratification of MB Patient Survival Rate by Gene Expression

GlioVis portal and the Cavalli dataset [20] were used to stratify MB patient overall survival rate by Ca^2+^ regulators expression using the Kaplan Meier method. Median expression was used as a cut-off threshold to assign their high (indicated in red) and low (indicated in blue) expression scores to MB patients for each gene of interest. A log-rank test was used to compare between the high and the low expression groups. The hazard ratios (HR) with 95% confidence interval (CI), for each gene of interest, are shown in Kaplan Meier figures. Data were extracted from GlioVis and plotted in GraphPad Prism.

### 2.7. Statistical Analysis

Statistical data analysis, scatter dot plots and heatmaps were performed and generated by GraphPad Prism software. Non-parametric statistical analysis was conducted using Kruskal Wallis multiple comparisons test (to compare gene expression differences between four MB subgroups and normal tissue). A two-tailed unpaired Mann Whitney test was used for statistical comparisons between two groups (MB and normal brain tissues studies, as well as metastasis analysis). For survival analysis, Gehan Breslow Wilcoxon and log-rank tests were used to compare between the high and the low score patient groups. All data are reported as mean with standard deviation, with *p*-values of <0.05 as statistically significant. Specific statistical tests and significance for each experiment are mentioned in the figure legends.

## 3. Results

### 3.1. Expression of Ca^2+^ Transporters in Normal Brain and MB Tissues

**Figure 1:**
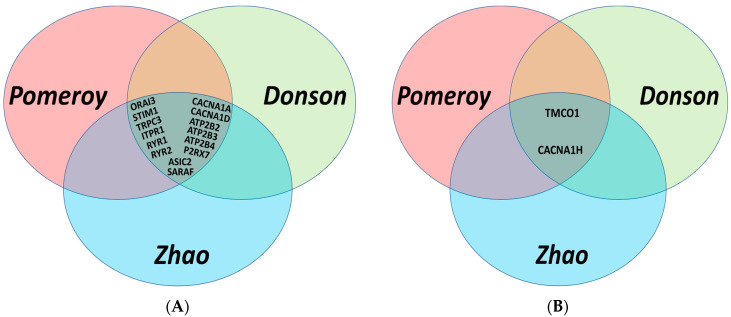
Venn diagrams illustrating the common genes with *p*-values < 0.01 that were differentially expressed in MB patients compared to normal brain tissues in three independent datasets; Pomeroy [17], Donson [18], and Zhao [19]: (**A**) Fourteen lower expressed genes in MB; (**B**) two higher expressed genes in MB.

Our first assessment was focused on studying the expression of 92 Ca^2+^ regulators in normal brain and MB tissues. To avoid dataset bias, we used three independent patient datasets, including Pomeroy [17], Donson [18] and Zhao [19] datasets. These studies demonstrated that out of the 92 genes, 14 genes were significantly downregulated, and two were significantly upregulated in MB tissues compared to normal brain tissues in all the three datasets (Table 2 and Figure 1). From the downregulated genes, three of them were plasma membrane Ca^2+^ pumps (ATP2B2, ATP2B3, ATP2B4), six were plasma membrane Ca^2+^ channels (CACNA1A, CACNA1D, P2RX7, TRPC3, ORAI3, ASIC2), and five were Ca^2+^ regulators located on the endoplasmic reticulum (ER) (ITPR1, RYR1, RYR2, STIM1, SARAF). From the upregulated genes, one was an ER Ca^2+^ channel, TMCO1, and one was the plasma membrane t-type Ca^2+^ channel, CACNA1H encoding the protein CaV3.2. A *p*-value of less than 0.01 was set as a cut-off for statistical significance for stringency in these analyses. TRPM2, TRPM3 and ITPR2, although showed a consistent pattern of downregulation levels in MB, compared to normal brain tissues, the *p*-values of these differences were not below 0.01 in all the three datasets and hence were not included in further studies.

### 3.2. Expression of Ca^2+^ Transporters in MB Molecular Subgroups

**Figure 2:**
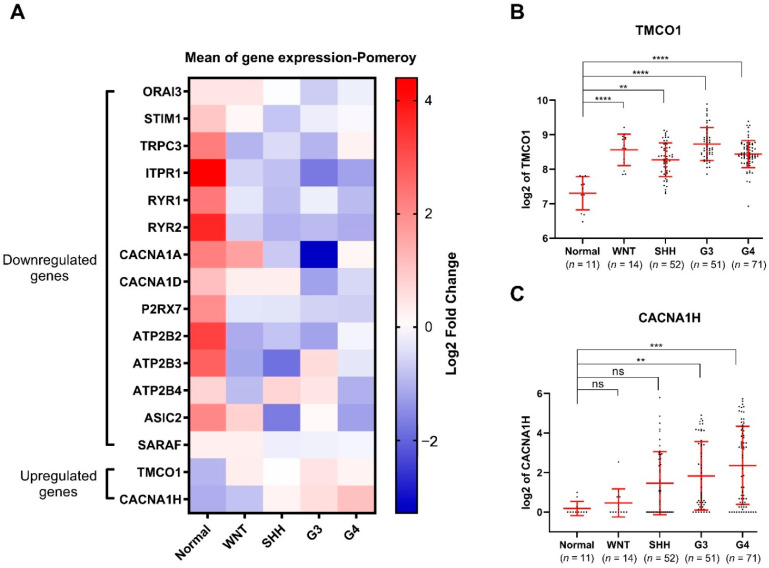
Expression of 2 upregulated and 14 downregulated Ca^2+^ transporter genes in MB subgroups compared to normal cerebellum present in the the Pomeroy dataset [17]: (**A**) Heatmap presenting the mean expression of the 16 identified Ca^2+^ transporter genes in MB subgroups compared to normal cerebellum; (**B**) TMCO1 and (**C**) CACNA1H mRNA expression in the Pomeroy dataset [17] via the R2 Genomics Analysis and Visualization Platform. The number of individuals in each group is listed below. ns: not significant *p* > 0.05, ** *p* < 0.01, *** *p* < 0.001, **** *p*-value < 0.0001, non-parametric test, with Kruskal Wallis test multiple comparisons, mean with SD.

Given these diagnostic and prognostic differences, it is of critical importance to conduct subgroup-specific studies. These analyses demonstrated that among the upregulated genes, TMCO1 is upregulated in all the four MB subgroups compared to the normal brain tissues (Figure 2A,B), while CACNA1H is upregulated in only aggressive Groups 3 and 4 subgroups (Figure 2A,C). Among the downregulated genes, TRPC3, ITPR1, RYR1, RYR2, P2RX7, ATP2B2, and ATP2B3 are down in all four subgroups, whilst the remaining genes are downregulated in specific subgroups (Figure S1 and Figure 2A).

### 3.3. Assessment of the Expression of Ca^2+^ Transporters in MB Metastasis

**Figure 3:**
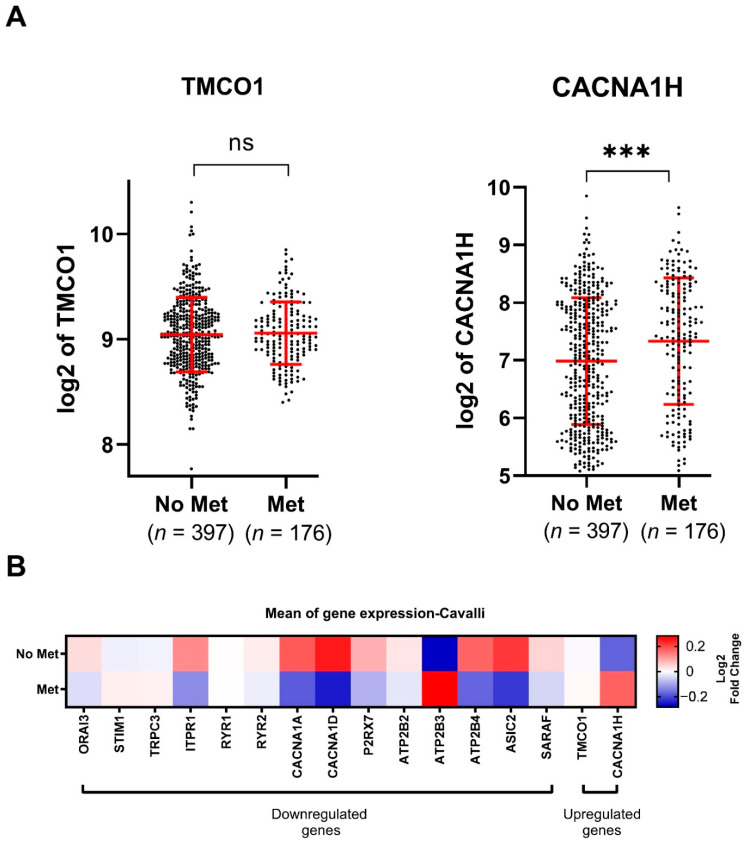
Expression of the identified dysregulated Ca^2+^ transporter genes in MB metastasis using the Cavalli dataset [20]: (**A**) TMCO1 and CACNA1H (2 upregulated Ca^2+^ transporter genes) log 2 transformed mRNA expression in the Cavalli dataset of MB metastatic patients (Met, *n* = 176) versus non-metastatic patients (No Met, *n* = 397). Data were exported from R2 Genomics software and plotted in GraphPad PRISM. ns: not significant *p* > 0.05, *** *p*-value < 0.001, two-tailed unpaired non-parametric *t*-test, with Mann Whitney test, mean with SD; (**B**) heatmap representing the mean expression of the 16 identified Ca^2+^ transporter genes in MB metastatic patients compared to MB non-metastatic patients.

To explore gene expression and its association with metastasis, we stratified the expression of the 16 dysregulated genes by patients with or without metastases. This revealed that TMCO1 expression did not show any association with the occurrence of metastasis (Figure 3A,B), whilst CACNA1H was significantly upregulated in tissues from metastatic patients (Figure 3B,C). Among the downregulated genes, lower levels of ORAI3, ITPR1, CACNA1D, P2RX7, ATP2B3, ATP2B4, ASIC2 and SARAF were significantly associated with the occurrence of metastasis, while the levels of ATP2B3 were positively correlated with metastasis occurrence (Figure S2 and Figure 3B). The expression for the remaining downregulated genes was not correlated to MB metastasis (Figure S2 and Figure 3B).

### 3.4. Stratification of MB Patient Survival by Expression of Ca^2+^ Regulators

**Figure 4:**
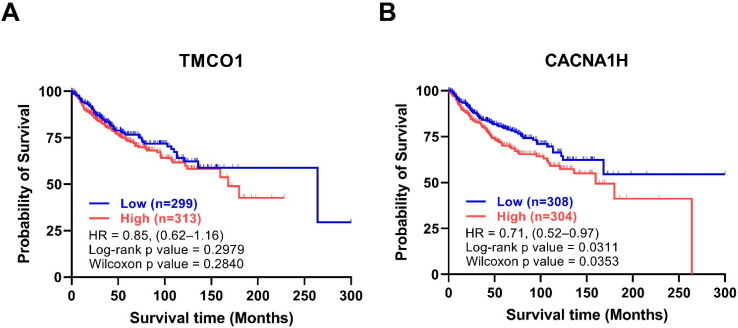
Kaplan Meier survival curves of the upregulated genes. Stratification of patient overall survival based on (**A**) TMCO1 and (**B**) CACNA1H expression in the Cavalli dataset [20]. Data were exported from GlioVis and plotted in GraphPad PRISM. The *Y*-axis represents overall survival probability, and the *x*-axis represents follow up in months, Blue: low expression, red: high expression, the number of patients of high and low expressions are shown between brackets, HR and *p*-values are shown in the graph, the total number of patients is 612.

To evaluate the association between the levels of the identified 16 genes and MB prognosis, the overall survival of 612 MB patients in the Cavalli cohort [20], using Kaplan-Meier Survival Curves, were assessed. Among the upregulated genes, while there was no significant association between TMCO1 levels and MB survival (Figure 4A), MB patients with high CACNA1H levels showed significantly reduced overall survival rates (Figure 4B). Among the downregulated genes, patients with low levels of CACNA1A and CACNA1D showed significantly lower survival rates (Figure S3). Table 3 summarises the association of the expression level of the 16 Ca^2+^ regulator genes with MB molecular subgroups and metastasis and survival rates.

### 3.5. Expression of the Ca^2+^ Regulating Genes in a Spontaneous MB Mice Model

**Figure 5:**
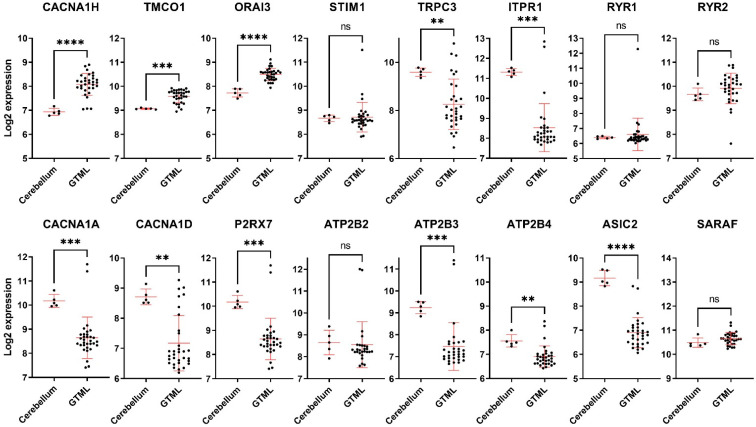
Expression of the dysregulated Ca^2+^ transporter genes in GTML mice. mRNA expression (in log2) of 5 normal cerebellum and 32 MB tumours from the GTML mouse model, sourced from the GEO dataset with accession number GSE36594. ns: not significant *p* > 0.05, ** *p* < 0.01, *** *p* < 0.001, **** *p*-value < 0.0001, two-tailed unpaired non-parametric *t*-test, with Mann Whitney test, mean with SD.

Given that preclinical studies are conducted in animal models, it is critical to assess the correlation between laboratory models and patient observations. Hence, we assessed whether the observed alterations in Ca^2+^ regulating genes in MB patients are also seen in an in vivo MB laboratory model. Tet-inducible Glt1-tTA; TRE-MYCN/Luciferase (GTML) is a transgenic model of MB driven by the expression of MYCN [22]. The GTML model gives rise to tumours that mostly (>80%) resemble Group 3 MB but also smaller sets of WNT, SHH, and Group 4 [22,23]. Our studies showed that, similar to patient data, the expression of upregulated genes CACNA1H and TMCO1 is increased in GTML tumour tissues compared to the control mice cerebellum tissues (Figure 5). Of the 14 downregulated genes, 8 genes (TRPC3, ITPR1, CACNA1A, CACNA1D, P2RX7, ATP2B3, ATP2B4, and ASIC2) showed a similar pattern with lower expression levels in GTML tumour tissues, while ORAI3 was the only gene that showed an opposite pattern with higher expression in GTML tumour tissues compared to the control mice tissues (Figure 5). The rest of the genes did not show any statistically significant difference (Figure 5). In addition, to the 16 altered genes, we also assessed the expression of the remaining original 92 genes in the GTML model (Figure S4). These analyses showed considerable differences in the expression of Ca^2+^ transporter genes between human MB patients and the GTML mouse model. Indeed, ~80% of genes that showed no statistically significant difference in the human MB patient analysis were altered significantly in the GTML tumour compared to normal cerebellum tissues (Figure S4).

### 3.6. Expression of the CACNA1 Genes in Different Brain Sections

**Figure 6:**
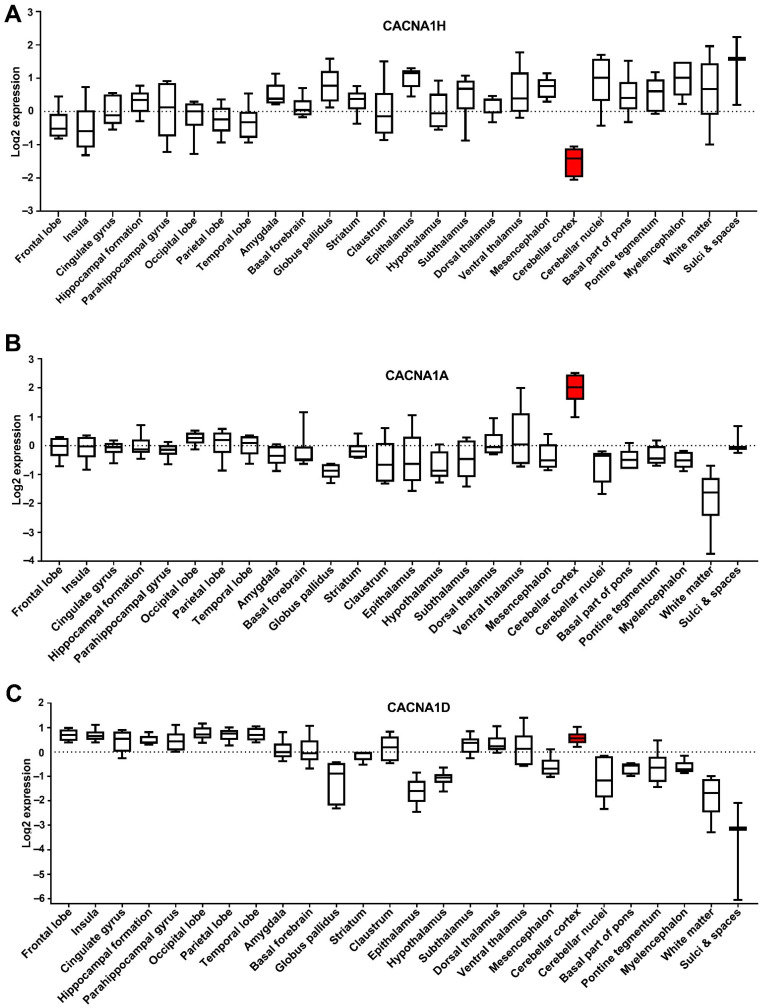
Expression of the CACNA1 genes in different brain sections. Microarray data from 6 brain samples obtained from the Allen Human Brain Atlas dataset, showing the expression of (**A**) CACNA1H, (**B**) CACNA1A and (**C**) CACNA1D in log2. Box and Whiskers plot (whiskers indicate min to max) is shown. Cerebellar cortex box is highlighted in red.

**Figure 7:**
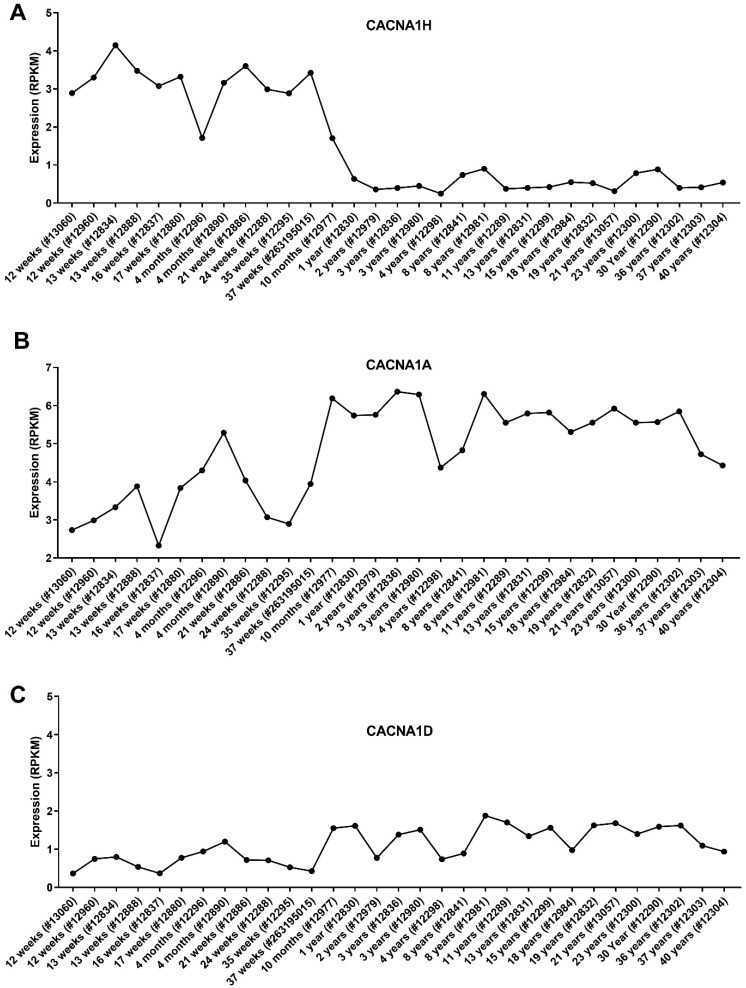
Expression of the CACNA1 genes in the cerebellum of individuals of different ages. Microarray data of the cerebellum of the developing brain were obtained from the BrainSpan Atlas of the Developing Human Brain. A total of 31 cerebellum samples from 12 post-conceptual weeks to 40 years. Expression values of (**A**) CACNA1H, (**B**) CACNA1A and (**C**) CACNA1D are shown in RPKM. Donor ID for each sample is stated in the bracket.

Cancer utilises developmental processes during the initiation and progression of tumours, particularly paediatric cancers [24]. Genes are often deregulated in the tissue of origin for a cancer, and this may provide insight into the role they play in tumorigenesis. We assessed the expression of the dysregulated Ca^2+^ genes in different parts of the brain using the Allen Brain Atlas dataset (www.brain-map.org (accessed on May 2021)). We focused on the three identified CACNA1 genes; CACNA1H, CACNA1A and CACNA1D, given their association with MB patient survival and metastasis rates. Intriguingly, CACNA1H, as one of the significantly upregulated genes in MB, showed the lowest level of expression in the cerebellar cortex, an origin site of MB (Figure 6A). In contrast, CACNA1A (a downregulated gene in MB) showed its highest expression in the cerebellar cortex (Figure 6B). The expression of CACNA1D, another downregulated gene in MB, in the cerebellar cortex was also among the high expressing brain sections (Figure 6C). Given that these analyses were conducted in datasets that include individuals from 24 to 55 years old, we thought to assess the expression of these genes in the cerebellum of individuals of different ages from infancy to adults. This analysis is particularly important given the increased occurrence of MB in children and the potential impact of the developing brain in MB progression. We, therefore, used the Atlas of the Developing Human Brain dataset, which that includes expression profiles of the cerebellum in individuals from 12 weeks to 40 years old (www.brainspan.org (accessed on May 2021)). Interestingly, these data showed that CACNA1H levels in the cerebellum show a clear decrease in individuals from 10 months onwards (Figure 7A). In contrast, CACNA1A shows an upward expression trajectory correlating with brain age, whilst CACNA1D levels remained steady (Figure 7B,C).

## 4. Discussion

Calcium signalling regulates several cancer hallmarks, including angiogenesis, metastasis, cell proliferation, and resistance to cell death [25,26]. Altered expression and/or activity of calcium regulator proteins have been reported in several cancers [25,27], and modulators of specific Ca^2+^ regulating proteins have been proposed to represent promising future therapeutic agents [28,29]. In this study, we assessed the expression of calcium regulator genes in MB, a cancer for which calcium signalling pathways are a highly underexplored area of research. This is while, for some other types of brain cancers, targeting calcium signalling has been proposed as a potential therapeutic approach, in particular in glioblastoma [30].

**Figure 8:**
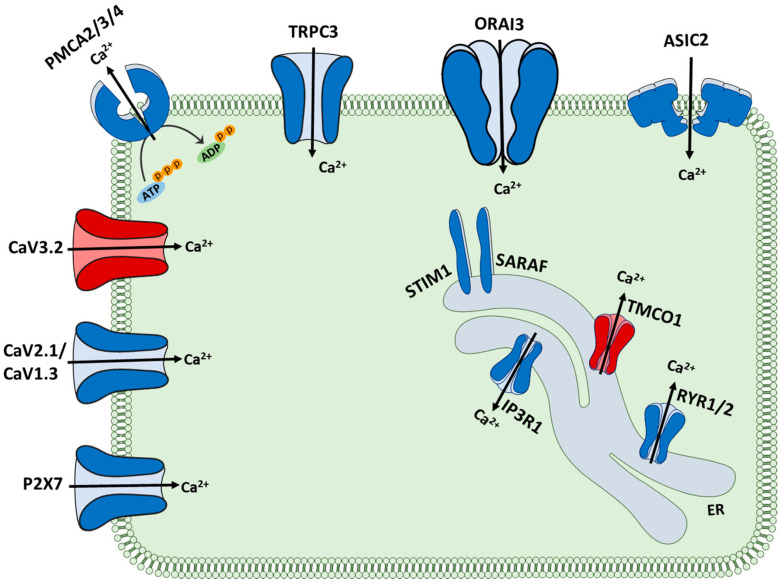
Schematic representation of the 16 Ca^2+^ regulators with altered expression in MB compared to normal brain. The 2 regulators with upregulated expression are shown in red, while the 14 regulators with downregulated expression are shown in blue.

In this study, we identified consistent alteration in the expression of 16 calcium regulators in medulloblastoma in three independent datasets. Of these, 14 were downregulated, and 2 were upregulated in MB compared to normal brain tissues. These Ca^2+^ related genes are differentially localised and perform different functions in Ca^2+^ metabolism (Figure 8). There is a decrease in the level of 6 plasma membrane calcium channels, including ASIC2, ORAI3, TRPC3, CaV2.1, CaV1.3 and P2X7. While this may result in a low level of Ca^2+^ influx, the expression of plasma membrane Ca^2+^ pumps, PMCA2, PMCA3 and PMCA4 are also decreased, potentially resulting in the maintenance of intracellular Ca^2+^ levels. The levels of the ER channels, RYR1, RYR2 and ITPR1 are decreased, potentially resulting in overloading of the ER calcium levels. This may explain the observed increase in TMCO1 gene expression that may be able to circumvent ER Ca^2+^ overload. Three regulators involved in the store-operated Ca^2+^ entry (SOCE), including ORAI3, STIM1 and SARAF, demonstrate lower expression levels in MB tissues. This may result in a decreased activity of the SOCE pathway in MB cells. Interestingly, alteration in the expression and/or activity of the SOCE components has been shown in multiple cancers. In breast cancer, elevated ORAI1 and lower ORAI3 levels are features of basal breast cancers [31], and in prostate cancer cells, reduced SOCE confers resistance to some apoptotic pathways [32]. It should be noted that to understand the involvement of Ca^2+^ signalling pathways in MB, in addition to gene expression studies, the assessment of protein expression and activity of Ca^2+^ transporters is of critical importance. Furthermore, calcium signalling is also regulated and influenced by synergistic interplay between different ions, including potassium. For instance, potassium-stimulated depolarization facilitates Ca^2+^ entry through T-type (low depolarization potential) or L-type (higher depolarization potential) channels [33]. On the other hand, calcium-activated potassium channels (BK channels), stimulated by Ca^2+^ or Mg^2+^, are responsible for the majority of K^+^ conductance throughout the cell membrane [34,35]. In MB, voltage-gated potassium channel EAG2 is upregulated in human-derived MB cells, where it promotes tumour growth in vitro [36,37]. In contrast, BK channels are reported to be diminished in medulloblastoma cell lines in their expression and activity [38].

The initial assessment was conducted on 92 Ca^2+^ regulating genes, including calcium pumps, channels, receptors, and sensors. Many of these genes are druggable, and exploring their relevance in MB could have an impact on future therapeutic options. Although proteins encoded by these genes are directly involved in the mobilisation of Ca^2+^, their activities can be regulated through their partner proteins. Hence, future studies can assess the expression of partner proteins for different Ca^2+^ transporters to further understand how Ca^2+^ signalling pathways are regulated in MB. Gene expression analyses in this study will allow the formation of hypotheses to focus on certain genes for future studies. These data should be complemented with laboratory-based experimental data to draw conclusive remarks about the role of calcium signalling pathways in MB initiation and progression, and the potential of targeting these pathways for MB treatment. Recent developments in the generation of murine models of MB [39], will have a significant influence on our understanding of MB. In this line, it would be interesting to isolate neurons or produce induced pluripotent stem cells (iPSCs) from the GTML mice and assess calcium efflux/influx levels in differentiated neurons to identify whether there are differences in pre-neoplastic tissues prior to tumour formation.

MB, like many other instances of cancer consist of multiple diseases, with four distinct molecular subgroups for MB tumours including WNT, SHH, Group 3 and Group 4. MB tumours frequently metastasise and spread to the spinal and intracranial spaces. Group 3 and 4 MBs show the highest levels of metastasis at diagnosis with ~47% and ~30% respectively [40]. MB metastasis occurs through leptomeningeal dissemination, where the tumour cells spread through the cerebrospinal fluid to the leptomeninges of the brain and spinal cord [41,42]. Although metastasis is responsible for 100% of MB-related deaths, it is the most unknown part of MB pathogenesis [42] and understanding this critical process and the genes involved is of high importance. Our analysis demonstrated that some calcium genes are generally deregulated in cancer, whilst others show subtype-specific deregulation. Additionally, some of these calcium genes were also associated with the incidence of metastasis, including CACNA1H, ORAI3, ITPR1, CACNA1D, P2RX7, ATP2B4, ASIC2, ATP2B3 and SARAF. Furthermore, the expression of three genes (CACNA1H, CACNA1A, CACNA1D) is significantly correlated with the overall survival of MB patients. Interestingly, both CACNA1H and CACNA1D were correlated with metastasis and patient survival rates. CACNA1H was overall upregulated in aggressive Groups 3 and 4 MB; however, two distinct populations of low and high expressions clearly existed. A similar trend was also observed in the metastasis analysis, although there was an overall association between higher CACNA1H levels and increased metastasis rate, a considerable variability in expression in the metastatic patients could be observed. Hence, CACNA1H expression alone does not appear to be sufficient to mediate metastasis and may be more critical for other cancer-associated processes. Stratifying CACNA1H expression revealed that patients with higher CACNA1H levels show lower survival rates, suggesting its importance in tumour aggressiveness or treatment response.

CACNA1D, on the other hand, showed an opposite expression pattern with its expression being downregulated in Groups 3 and 4 and its higher expression associated with lower metastasis and higher survival rates. CACNA1A, the other CACNA1 gene with altered expression in MB, also showed a similar expression pattern as a CACNA1D gene with lower expression in Group 3 and 4 MB and a reverse association of its expression level with patient survival rate.

CACNA1H, CACNA1D and CACNA1A encode for CaV3.2, CaV1.3 and CaV2.1 proteins, respectively. Intriguingly, our previous studies of screening plasma membrane calcium channel modulators against MB cell growth, identified mibefradil and NNC55-0396, two inhibitors of the CaV3 channels, to suppress 3D growth of MB cells [43]. Our exploration of CACNA1H expression patterns in this current study and our previous data with CaV3 inhibitors, may suggest an important role of these calcium channels in MB progression. Inhibition of CaV3 channels with mibefradil was also previously reported to suppress the growth of glioblastoma stem-like cells and increase their sensitivity to the chemotherapeutic agent, temozolomide [44]. Importantly, a recent Phase I clinical trial for recurrent high-grade glioma showed that mibefradil in combination with temozolomide was well-tolerated in patients and showed no toxicity [45] (ClinicalTrials.gov identifier NCT01480050). Further studies are warranted to understand the role of CaV channels in MB and the possibility of harnessing them to control MB progression.

Intriguingly, we showed that CACNA1H expression levels in the cerebellum show a clear decrease with age, while CACNA1A levels show an opposite trajectory. This is an important observation given that MB is thought to arise from disruptions in cerebellar development [46] and the role of calcium transients in the regulation of various stages of neuronal development [47]. Indeed, gain-of-function mutations in the CACNA1H gene via signalling Ca^2+^-regulated transcription factors disrupt neuronal development, resulting in an increase in seizure susceptibility in epilepsy patients [48].

Our studies did not show any difference in the gene expression of TRPC4, while previous studies by Wei et al. showed that activation of the TRPC4 calcium channel increased MB cell motility [15]. Wei et al. study, however, did not focus on a conclusive comparison between the expression of healthy brain and MB tissues. In addition, involvement of Ca^2+^ transporters in disease pathology can be due to their altered activity or localisation and not necessarily a change in their expression. Indeed, signals from some of the plasma membrane Ca^2+^ channels regulate the expression or activity of proteins involved in cancer-associated processes such as cell migration and epithelial to mesenchymal transition [49].

Our studies also identified that from the 16 altered genes, 10 genes show the same patterns of expression in the GTML mouse model of MB. Orai3 was the only gene to display an opposite expression pattern. However, considerable differences in the gene expression patterns of the remaining genes from the original 92 genes were observed between the human MB patients and GTML mice model. These differences could be due to the fact that the GTML model mainly (>80%) presents Group 3 MB [22,23], while MB human patient samples were of diverse molecular subgroups as shown in the subgroup studies. Furthermore, overall, more variabilities in the expression of each gene in between individuals in human patient samples could be observed, while these expression variabilities were considerably less in GTML model. This could clearly affect the statistical test outcomes and can point towards intrinsic differences between a laboratory-made animal model and human patient samples. It is, therefore, critical not to only rely on the GTML mice model, at least in regard to calcium signalling studies. More advanced and specific MB mice models are also needed in MB research. One such model is a model that is being recently developed that accurately and specifically recapitulates aggressive Group 3 MB [50].

## 5. Conclusions

In conclusion, this study demonstrated deregulation of gene expression for calcium signalling regulators in MB patients. Of the many genes involved and associated with calcium signalling, this study highlights those that are of highest priority in future studies, that aim to understand the role of calcium signalling in medulloblastoma patients and whether there are potential therapeutic avenues targeting this signalling. In silico gene expression analyses in this study will allow the formation of hypotheses to focus on certain genes, however laboratory-based experimental data are warranted to draw conclusive remarks. These include the assessment of protein expression and activity of Ca^2+^ transporters in MB, as well as the effect of dysregulation of their activities in MB initiation and progression. Furthermore, this study focused on 92 Ca^2+^ regulating genes, including calcium pumps, channels, receptors, and sensors. Given that Ca^2+^ signals are also regulated by partner proteins of some these Ca^2+^ transporters, future studies can assess the expression of these partner proteins to further understand the regulation of Ca^2+^ signalling pathways in MB.

## Figures and Tables

**Table 1:** A brief description of the four datasets used for this study.

Author	No. of Samples	No. of MB Samples	No. of Normal Samples	Platform	Ref.
Pomeroy	204	188	11	u133a	[17]
Donson	130	22	13	u133p2	[18]
Zhao	92	18	12	ilmnhwg6v2	[19]
Cavalli	763	763	0	hugene11t	[20]

**Table 2:** The average mRNA (log 2) expression and *p*-values of Ca^2+^ transporter genes in MB patient tumour tissues and normal brain tissues (N) in three independent datasets; Pomeroy [17], Donson [18], and Zhao [19]. Highlighted genes in blue show significantly lower expression in MB tissues (compared to normal brain tissue) in all the three datasets, whilst genes highlighted in red, higher expressed genes. The *p*-values were calculated through a two-tailed unpaired non-parametric *t*-test (Mann-Whitney test). A *p*-value of less than 0.01 was set as a cut-off for statistical significance.

Gene	Average of mRNA Expression (Log 2) and *p* Values								
	Pomeroy			Donson			Zhao		
	N	MB	*p*-Values	N	MB	*p*-Values	N	MB	*p*-Values
Orai1	- ^1^	-	-	5.891	5.860	0.5495	3.992	4.881	0.0575
Orai2	6.429	6.092	0.2284	8.434	8.686	0.2459	5.258	4.933	0.5451
Orai3	4.643	3.942	0.0054	6.806	5.871	0.0005	5.344	2.779	<0.0001
STIM1	5.124	3.852	<0.0001	6.255	5.578	0.0012	8.080	6.803	<0.0001
STIM2	-	-	-	6.919	7.245	0.1414	7.809	7.502	0.1887
TRPC1	6.085	6.267	0.2886	7.774	7.636	0.6432	8.674	7.953	0.0016
TRPC3	6.165	3.519	<0.0001	5.234	3.971	0.0067	7.597	4.322	<0.0001
TRPC4	2.154	2.208	0.9947	2.704	1.151	0.0048	4.529	4.456	0.5948
TRPC5	3.479	3.389	0.8723	2.724	2.444	0.5158	4.813	4.502	0.1014
TRPC6	1.727	1.940	0.7644	2.545	3.040	0.5159	1.810	0.468	0.0007
TRPV1	5.540	5.202	0.3499	5.845	5.667	0.7941	6.410	5.942	0.0491
TRPV2	1.555	2.326	0.1314	5.445	4.360	0.0003	5.123	4.248	0.0664
TRPV3	-	-	-	2.992	3.578	0.1460	3.705	2.973	0.0098
TRPV4	3.641	2.812	0.0221	2.998	2.503	0.1885	5.405	6.802	0.1817
TRPV5	1.815	1.516	0.4167	3.135	3.694	0.2530	3.219	1.609	0.0022
TRPV6	2.715	2.925	0.2729	2.789	2.239	0.4527	3.330	1.549	0.0146
TRPA1	1.948	2.375	0.2922	1.818	1.464	0.5659	1.798	1.976	0.9253
TRPP2	6.658	6.628	0.8891	7.562	7.832	0.4946	7.395	6.807	0.0033
TRPP3	0.979	1.746	0.0365	5.542	3.798	<0.0001	0	2.199	0.0109
TRPP5	3.391	2.236	0.0066	1.632	2.112	0.2456	0	0	>0.9999
TRPML1	4.897	4.866	0.7630	7.228	6.965	0.1414	8.533	8.167	0.0016
TRPML2	-	-	-	2.176	3.328	0.0098	2.494	2.741	0.5176
TRPML3	1.123	1.629	0.2707	1.014	1.895	0.0541	0	0	>0.9999
TRPM1	0.999	1.022	0.9252	1.082	1.116	0.8989	0	0.114	0.5034
TRPM2	4.847	3.328	<0.0001	6.599	4.440	<0.0001	1.729	0.808	0.0201
TRPM3	9.098	7.089	0.0095	8.692	7.268	0.0410	6.649	5.436	0.0201
TRPM4	3.100	2.528	0.2973	3.847	2.052	0.0074	6.593	6.304	0.2900
TRPM5	-	-	-	0.669	0.532	0.3219	0.068	0.736	0.4860
TRPM6	1.084	1.170	0.9880	4.412	3.082	0.0014	4.974	4.147	0.0004
TRPM7	-	-	-	5.531	6.345	0.0002	5.434	5.248	0.2711
TRPM8	2.263	1.629	0.1311	2.715	3.418	0.2460	0	0.368	0.5034
ITPR1	10.088	4.475	<0.0001	9.773	4.813	<0.0001	10.483	5.481	<0.0001
ITPR2	4.973	4.323	0.0126	6.757	4.872	<0.0001	6.697	3.508	<0.0001
ITPR3	1.125	2.138	0.0677	3.227	4.622	0.0222	6.458	6.997	0.8268
RYR1	5.688	2.687	<0.0001	6.311	2.906	<0.0001	6.291	3.974	<0.0001
RYR2	7.446	2.840	<0.0001	7.293	2.959	<0.0001	7.862	3.736	<0.0001
RYR3	4.855	4.635	0.4665	7.978	5.471	<0.0001	2.499	1.959	0.1019
TMCO1	7.305	8.483	<0.0001	8.950	9.700	0.0075	9.836	10.666	<0.0001
TMCO2	-	-	-	2.098	2.537	0.4128	0.142	0.325	0.9717
TMCO3	4.555	3.731	0.0029	8.432	9.124	0.0042	10.508	9.555	<0.0001
TMCO4	-	-	-	2.968	1.423	0.0016	4.207	3.737	0.0128
TMCO5	-	-	-	3.860	3.738	0.7944	2.914	3.571	0.0375
TMCO6	4.864	5.253	0.0388	5.317	6.123	<0.0001	-	-	-
CACNA1A	11.316	8.204	<0.0001	9.878	8.134	0.0006	10.158	5.498	<0.0001
CACNA1B	0.590	1.988	0.0025	8.740	7.680	<0.0001	4.025	4.234	0.6389
CACNA1C	3.626	2.975	0.1255	7.329	5.360	<0.0001	6.936	6.233	0.0223
CACNA1D	5.434	3.875	<0.0001	6.145	3.693	<0.0001	4.263	3.163	<0.0001
CACNA1E	1.128	1.506	0.4616	7.416	6.044	0.0005	7.714	6.206	0.0199
CACNA1F	0.311	1.492	0.0102	2.662	2.357	0.2825	6.287	7.099	0.0101
CACNA1G	6.602	5.525	0.1339	5.832	4.881	0.0863	8.976	8.502	0.2037
CACNA1H	0.185	1.830	0.0004	1.812	3.948	0.0022	3.872	7.055	0.0001
CACNA1I	4.920	2.993	0.0003	7.012	4.645	<0.0001	1.181	1.483	0.4301
CACNA1S	0.996	1.736	0.3773	4.037	3.457	0.1885	0.866	4.521	0.0001
CATSPER1	-	-	-	2.975	3.842	0.1071	4.679	4.072	0.0003
CATSPER2	8.142	5.279	<0.0001	6.176	5.670	0.0929	7.983	3.176	<0.0001
CATSPER3	-	-	-	3.500	4.048	0.1036	2.987	3.284	0.2665
CATSPERB	2.241	1.898	0.4435	2.775	2.501	0.3146	0	0.100	>0.9999
CATSPERG	2.407	1.512	0.0326	2.092	2.047	0.5159	5.938	3.143	0.0005
P2RX1	2.469	2.011	0.2438	1.888	2.208	0.6790	4.128	4.091	0.6691
P2RX2	2.195	1.181	0.0036	3.202	3.872	0.0832	4.905	4.412	0.0016
P2RX3	0.148	0.689	0.0505	1.039	1.803	0.0978	4.506	4.634	0.5659
P2RX4	6.854	3.966	<0.0001	6.215	5.942	0.5495	9.206	6.718	<0.0001
P2RX5	5.106	2.694	<0.0001	6.760	3.673	<0.0001	0.116	0.308	0.7732
P2RX6	2.010	2.081	0.8929	4.548	4.318	0.4130	1.023	0.492	0.0933
P2RX7	4.039	1.556	<0.0001	7.652	4.099	<0.0001	7.597	2.043	<0.0001
ATP2A1	1.569	1.632	0.9713	2.984	3.801	0.1111	0	0.099	>0.9999
ATP2A2	10.370	9.189	<0.0001	9.996	9.949	0.9530	11.784	10.651	<0.0001
ATP2A3	6.050	2.523	<0.0001	3.310	3.878	0.3528	5.238	3.776	<0.0001
ATP2B1	8.523	8.777	0.1404	9.221	9.142	0.5328	8.284	5.096	<0.0001
ATP2B2	10.429	6.519	<0.0001	10.955	7.360	<0.0001	10.793	6.598	<0.0001
ATP2B3	5.496	2.222	<0.0001	8.174	4.797	<0.0001	8.779	5.280	<0.0001
ATP2B4	7.422	6.530	0.0098	8.474	7.562	0.0056	9.558	8.600	0.0005
ATP2C1	6.804	7.494	0.0002	8.287	8.332	0.7045	8.242	8.489	0.0969
ATP2C2	1.095	1.613	0.3845	3.802	3.765	0.9798	-	-	-
SLC24A6	-	-	-	5.572	5.610	0.7047	7.965	7.487	0.4210
CCDC109A	-	-	-	7.481	6.717	0.0002	8.759	6.366	<0.0001
ASIC1	6.197	6.868	0.0151	7.455	8.106	0.1194	9.507	9.987	0.2529
ASIC2	5.476	2.543	<0.0001	7.282	4.012	<0.0001	9.817	6.913	<0.0001
ASIC3	4.229	4.092	0.4391	4.997	4.613	0.7428	7.203	6.012	0.0001
ASIC4	2.518	2.367	0.6637	4.605	2.245	0.0003	7.748	7.373	0.0022
ASIC5	-	-	-	1.801	2.275	0.2389	3.408	3.221	0.9584
Piezo1	5.620	4.539	0.0140	6.792	6.139	0.1915	8.530	7.569	0.4089
Piezo2	2.396	3.588	0.0345	5.902	4.990	0.0401	5.458	4.846	0.1556
SARAF	10.925	10.505	0.0012	11.404	10.498	<0.0001	12.233	11.775	<0.0001
P2Y1	1.956	2.719	0.0994	3.035	3.355	0.4893	1.853	2.733	0.4084
P2Y2	1.492	1.284	0.7624	2.147	1.518	0.1602	2.805	2.211	0.0490
P2Y4	3.316	3.043	0.2647	2.777	4.020	0.0149	4.695	4.343	0.0970
P2Y6	2.172	1.493	0.1263	4.528	3.079	0.0024	4.580	3.803	0.0007
P2Y11	-	-	-	-	-	-	6.918	7.406	0.0028
P2Y12	-	-	-	6.978	3.526	<0.0001	3.803	0.624	<0.0001
P2Y13	2.453	2.649	0.7427	4.838	2.925	<0.0001	6.407	3.644	<0.0001
P2Y14	4.203	4.774	0.0643	5.665	5.616	>0.9999	0	0	-

^1^ “-” means that the gene is not available in the dataset.

**Table 3:** Summary of the association of the expression level of the 16 Ca^2+^ regulator genes with MB molecular subgroups and metastasis and survival rates.

Genes	Expression Level in MB	Subgroups Specific				Metastasis	Overall Survival
		WNT	SHH	G3	G4	Patients with ↑ Gene Expression	
TMCO1	High (↑)	√	√	√	√	-	-
CACNA1H		-	-	√	√	↑	↓
ORAI3	Low (↓)	-	-	√	-	↓	-
STIM1		-	√	√	√	-	-
TRPC3		√	√	√	√	-	-
ITPR1		√	√	√	√	↓	-
RYR1		√	√	√	√	-	-
RYR2		√	√	√	√	-	-
CACNA1A		-	√	√	√	-	↑
CACNA1D		-	-	√	√	↓	↑
P2RX7		√	√	√	√	↓	-
ATP2B2		√	√	√	√	-	-
ATP2B3		√	√	√	√	↓	-
ATP2B4		√	-	-	√	↓	-
ASIC2		-	√	√	√	↓	-
SARAF		-	√	√	-	↓	-

√: associated with specific subgroup; “-”: not significant (*p* > 0.05); “↑”: high. “↓”: low.

## Data Availability

Data used in this study were obtained from publicly available datasets. Names of each dataset and platform used to extract data from are stated in Section 2.

## Supplementary Materials

The following are available online at https://www.mdpi.com/article/10.3390/genes12091329/s1, Figure S1: Expression data analysis for the selected downregulated Ca^2+^ transporter genes in MB subgroups (*n* = 188) compared to normal cerebellum (*n* = 11) present in the Pomeroy dataset [17], via R2 Genomics Analysis and Visualization Platform., Figure S2: Expression data analysis for the metastasis status of the selected downregulated Ca^2+^ transporter genes using the Cavalli dataset [20], via R2 Genomics Analysis and Visualization Platform., Figure S3: Kaplan Meier curves of the overall survival of patients for of the fourteen key downregulated genes from the Cavalli dataset [20] via GlioVis software. Figure S4: Expression of Ca^2+^ transporter genes (genes that were not significantly altered in the human MB patient samples) in GTML mice.
