# Supplementary material for: Calcium Signalling in Medulloblastoma: An In Silico Analysis of the Expression of Calcium Regulating Genes in Patient Samples
Source: Genes (Basel). 2021 Aug 27;12(9):1329. doi: 10.3390/genes12091329 (PMC8468187; doi:10.3390/genes12091329)
Supplement: Supplementary file 1 [file genes-12-01329-s001.zip › genes-1307304-supplementary.pdf]

Calcium signalling in medulloblastoma: an *in silico* analysis of the expression of calcium regulating genes in patient samples

Ahmed Maklad [1], Mohammed Sedeeq [1], Michael J. G. Milevskiy [2,3], Iman Azimi [1*]

Supplementary data

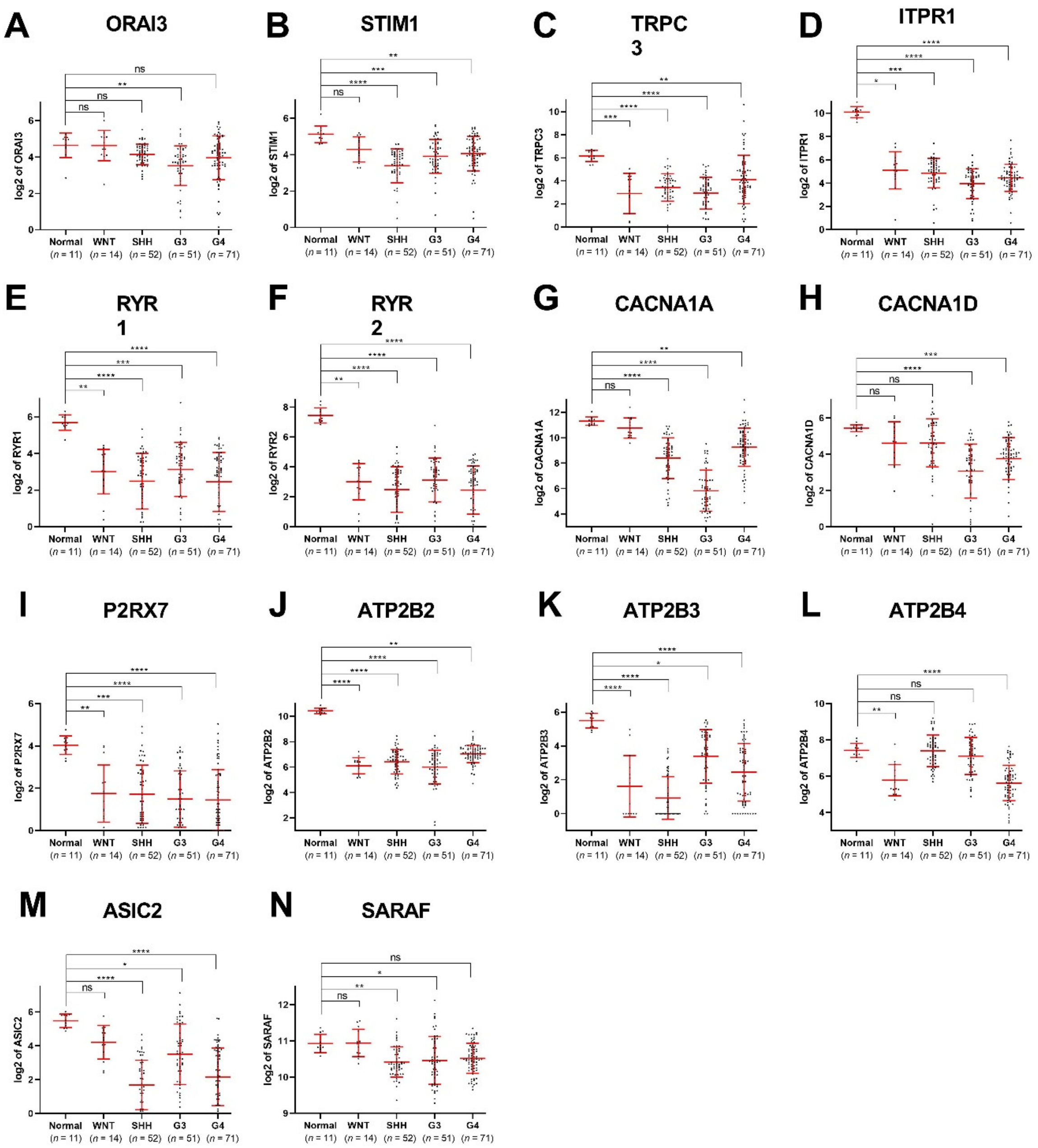

Figure S1. Expression data analysis for the selected downregulated Ca^{2+} transporter genes in MB subgroups (n = 188) compared to normal cerebellum (n = 11) present in *Pomeroy* dataset [1], via R2 Genomics Analysis and Visualization Platform: (A) ORAI3; (B) STIM1; (C) TRPC3; (D) ITPR1; (E) RYR1; (F) RYR2; (G) CACNA1A; (H) CACNA1D; (I) P2RX7; (J) ATP2B2; (K) ATP2B3; (L) ATP2B4; (M) ASIC2; (N) SARAF. (ns: not significant $P > 0.05$, *$P < 0.05$, ** $P < 0.01$, *** $P < 0.001$, **** P *value* < 0.0001, Non-parametric test, with Kruskal-Wallis test multiple comparisons, Mean with SD).

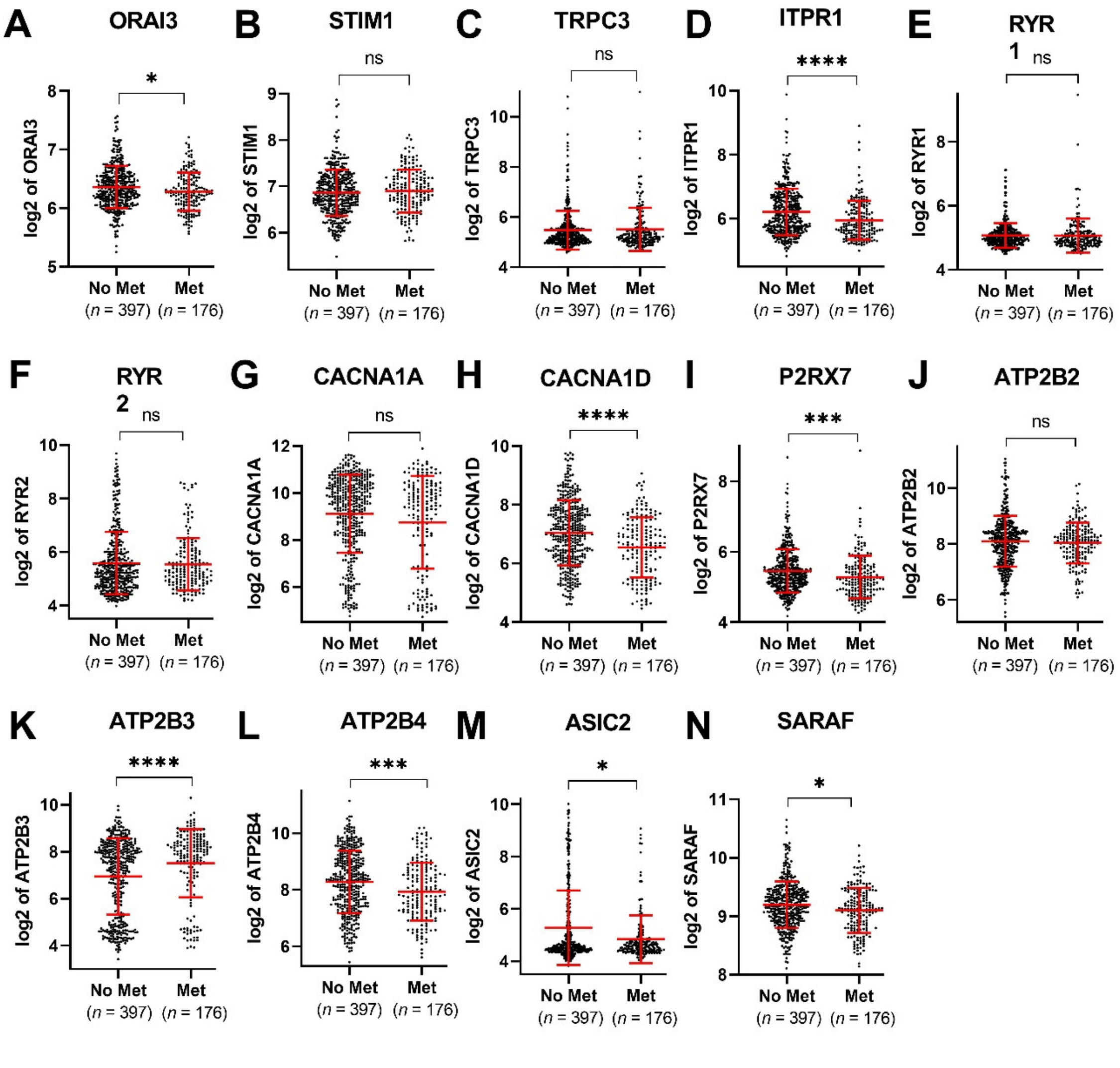

Figure S2. Expression data analysis for the metastasis status of the selected downregulated Ca^{2+} transporter genes using C*avalli* dataset [2], via R2 Genomics Analysis and Visualization Platform: (A) ORAI3; (B) STIM1; (C) TRPC3; (D) ITPR1; (E) RYR1; (F) RYR2; (G) CACNA1A; (H) CACNA1D; (I) P2RX7; (J) ATP2B2; (K) ATP2B3; (L) ATP2B4; (M) ASIC2; (N) SARAF. Log 2 transformed expression of MB Metastatic patients (Met, n = 176) versus non-metastatic patients (No Met, n = 397). (ns: not significant $P > 0.05$, *$P < 0.05$, *** $P < 0.001$, **** P *value* < 0.0001, two-tailed unpaired non-parametric t-test, with Mann-Whitney test, Mean with SD).

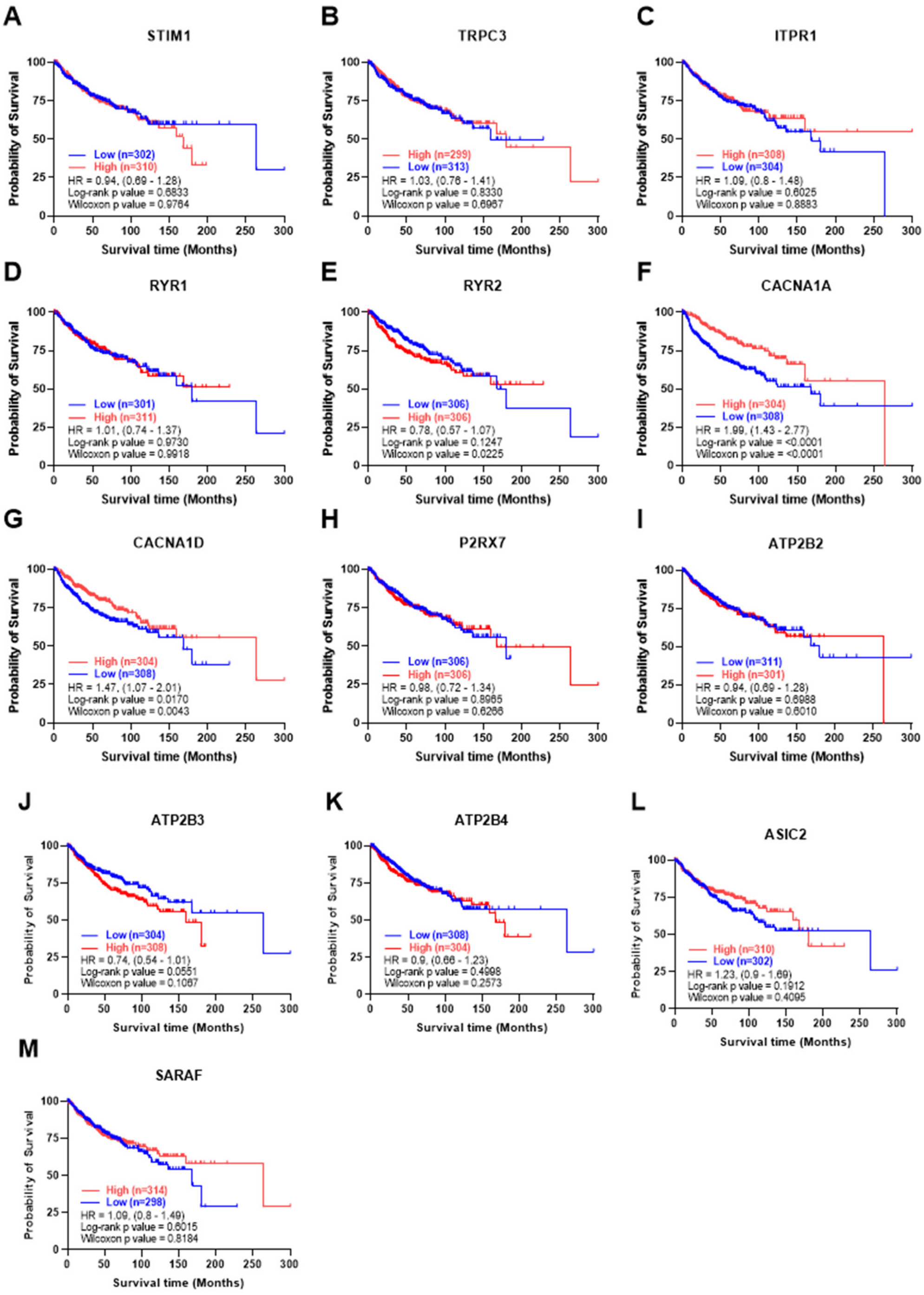

Figure S3. Kaplan-Meier curves of the overall survival of patients for of the fourteen key downregulated genes from the *Cavalli* dataset [2] via GlioVis software: (A) STIM1; (B) TRPC3; (C) ITPR1; (D) RYR1; (E) RYR2; (F) CACNA1A; (G) CACNA1D; (H) P2RX7; (I) ATP2B2; (J) ATP2B3; (K) ATP2B4; (L) ASIC2; (M) SARAF. where y-axis represents overall survival probability, and x-axis represents follow up in months, Blue: low expression, Red: high expression, the number of patients of high and low expressions are shown between brackets, *P value* is shown in the graph, the total number of patients used are 612. ORAI3 is not available in *Cavalli* dataset.

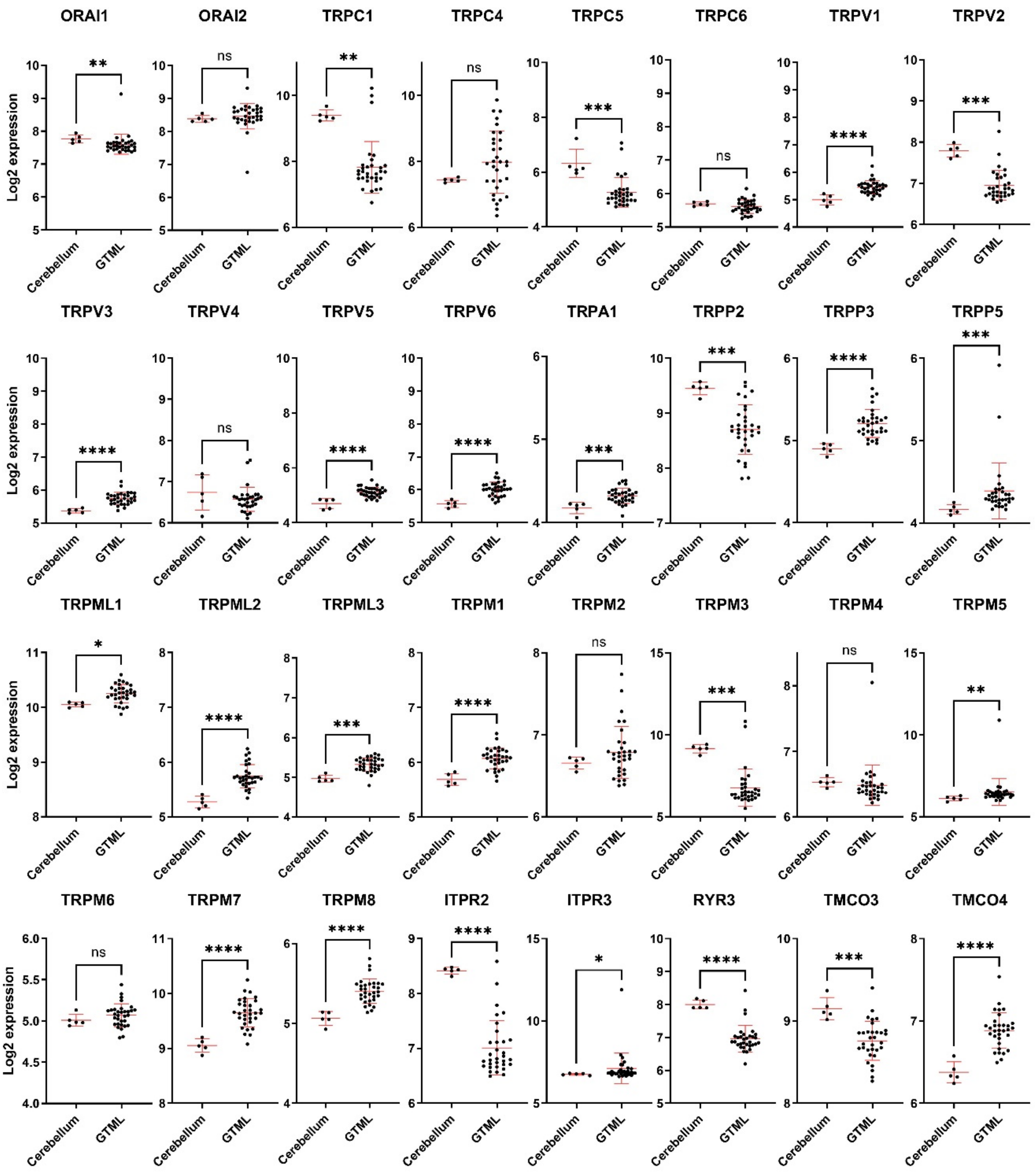
ORAI1
**
ORAI2
ns
TRPC1
**
TRPC4
ns
TRPC5
***
TRPC6
ns
TRPV1
****
TRPV2
***
TRPV3
****
TRPV4
ns
TRPV5
****
TRPV6
****
TRPA1
***
TRPP2
***
TRPP3
****
TRPP5
***
TRPML1
*
TRPML2
****
TRPML3
***
TRPM1
****
TRPM2
ns
TRPM3
***
TRPM4
ns
TRPM5
**
TRPM6
ns
TRPM7
****
TRPM8
****
ITPR2
****
ITPR3
*
RYR3
****
TMCO3
***
TMCO4
****
Log2 expression
Cerebellum
GTML

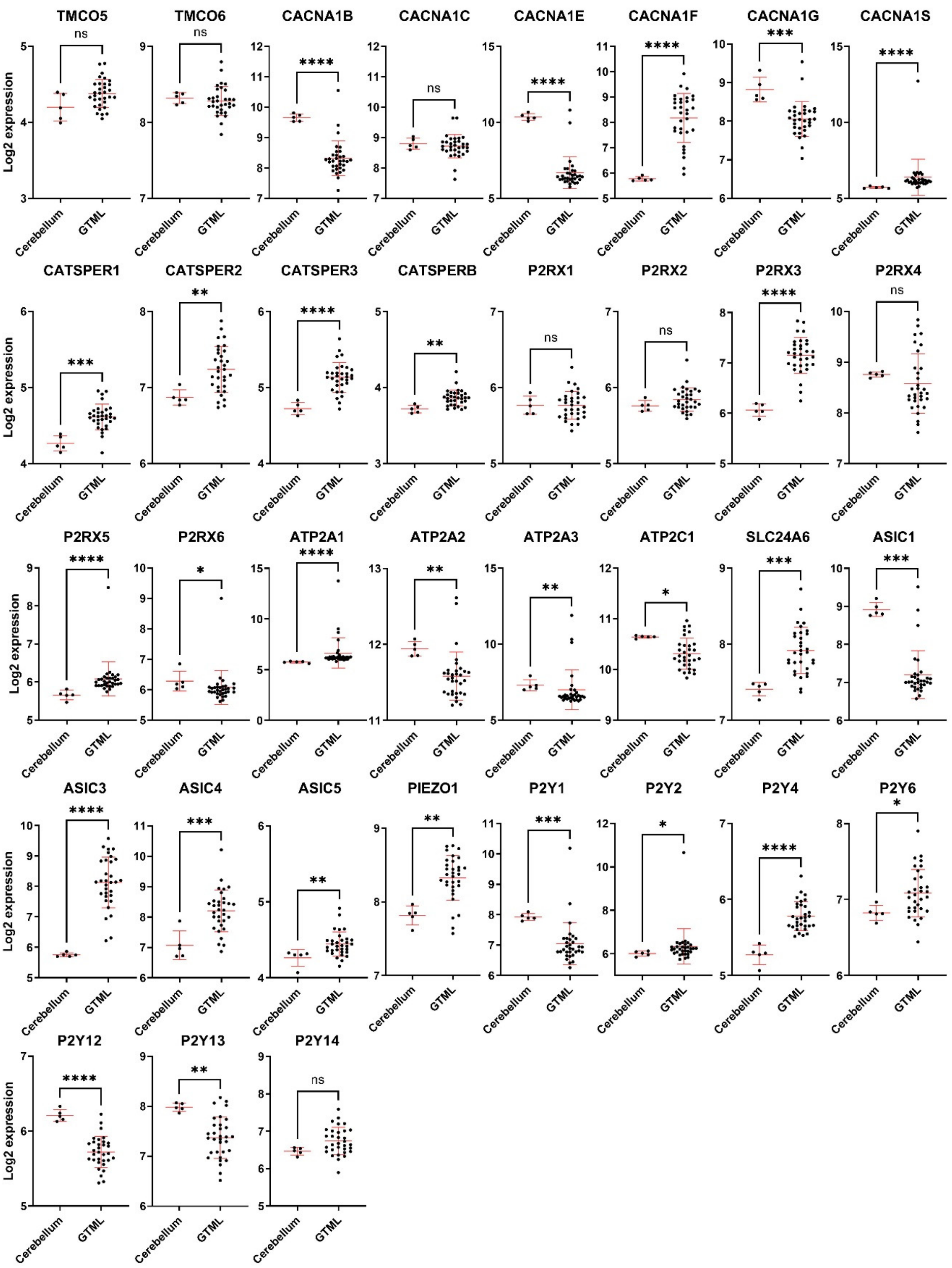

Figure S4. Expression of Ca^{2+} transporter genes (genes that were not significantly altered in the human MB patient samples) in GTML mice. mRNA expression (in log2) of 5 normal cerebellum and 32 MB tumours from the GTML mouse model, sourced from the GEO dataset with accession number GSE36594. Dataset did not include gene expression of STIM2, TMCO2, CACNA1I, ATP2B1, ATP2C2, CCDC109A, Piezo2, CATSPERG and P2Y11. ns: not significant $P > 0.05$, $^{**}P < 0.01$, *** $P < 0.001$, **** P value < 0.0001, two-tailed unpaired non-parametric t-test, with Mann-Whitney test, Mean with SD.

References

1. Cho, Y.-J.; Tsherniak, A.; Tamayo, P.; Santagata, S.; Ligon, A.; Greulich, H.; Berhoukim, R.; Amani, V.; Goumnerova, L.; Eberhart, C.G.; et al. Integrative genomic analysis of medulloblastoma identifies a molecular subgroup that drives poor clinical outcome. *J Clin Oncol* **2011**, *29*, 1424-1430, doi:10.1200/JCO.2010.28.5148.
2. Cavalli, F.M.G.; Remke, M.; Rampasek, L.; Peacock, J.; Shih, D.J.H.; Luu, B.; Garzia, L.; Torchia, J.; Nor, C.; Morrissy, A.S.; et al. Intertumoral Heterogeneity within Medulloblastoma Subgroups. *Cancer Cell* **2017**, *31*, 737-754.e736, doi:10.1016/j.ccell.2017.05.005.
